# The management of pediatric Chiari I malformation with concomitant hydrocephalus: a multicenter retrospective analysis

**DOI:** 10.1007/s00381-025-07052-4

**Published:** 2025-11-25

**Authors:** Ladina Greuter, Tim Jonas Hallenberger, Maria Licci, Ulrich-Wilhelm Thomale, Valentina Pennacchietti, Andrea Bartoli, Mahmoud Messerer, Agne Andriuskeviciute, Marta Garvayo, Malte Ottenhausen, Verena Fassel, Conor Mallucci, Arthur R. Kurzbuch, Giuseppe Cinalli, Guiseppe Mirone, Christopher Chandler, Sanjeev Bassi, Martina Messing-Juenger, Katharina Lutz, Dieter Class, Jonathan Roth, Shlomi Constantini, Carla Richetta, Raphael Guzman, Jehuda Soleman

**Affiliations:** 1 Department of Neurosurgery, University Hospital Basel, Basel, Switzerland; 2 Division of Pediatric Neurosurgery, Children’s University Hospital of Basel, Spitalstrasse 21, 4031 Basel, Switzerland; 3 Division of Pediatric Neurosurgery, Charité Universitätsmedizin Berlin, Berlin, Germany; 4 Department of Neurosurgery, University Hospital of Geneva, Geneva, Switzerland; 5 Department of Neurosurgery, University Hospital of Lausanne, Lausanne, Switzerland; 6 Department of Neurosurgery, University Hospital of Mainz, Mainz, Germany; 7 Department of Neurosurgery, Alder Hey Children’s NHS Foundation Trust, Liverpool, UK; 8 Department of Pediatric Neurosurgery, Santobono-Pausilipon Children’s Hospital, AORN, Naples, Italy; 9 Pediatric and Adult Neurosurgery, King’s College Hospital, NHS Trust, London, UK; 10 Department of Neurosurgery, Asklepios Children’s Hospital, Sankt Augustin, Germany; 11 Department of Neurosurgery, University Hospital Bern, Bern, Switzerland; 12 Department of Neurosurgery, University Hospital of Magdeburg, Magdeburg, Germany; 13 Department of Pediatric Neurosurgery, the Pediatric Brain Center, Dana Children’s Hospital, Tel Aviv University, Tel Aviv, Israel; 14 Faculty of Medicine, University of Basel, Basel, Switzerland

**Keywords:** Pediatric neurosurgery, Chiari I malformation, Hydrocephalus, Syrinx, Arnold Chiari, Endoscopic third ventriculostomy

## Abstract

**Background:**

Chiari I malformation (CIM) rarely occurs alongside hydrocephalus (7–10%), and the relationship between the two—whether one is the cause or the effect of the other—remains contentious. Consequently, the optimal treatment approach for these patients is unclear. This multicenter, retrospective study analyzed management strategies and outcomes for children presenting with CIM and hydrocephalus, with or without syringomyelia.

**Methods:**

We reviewed cases from 2005 to 2021 involving children (< 18 years) who underwent surgical treatment for CIM with hydrocephalus, with or without syringomyelia, at ten international pediatric neurosurgical centers. The primary outcome was the rate of revision surgery due to treatment failure, defined as the need for additional surgery due to unsatisfactory treatment of CIM and/or hydrocephalus by the initial intervention. Descriptive survival analyses and a step-down multivariable analysis to identify possible risk factors influencing the outcome were performed.

**Results:**

The study included 49 patients (mean age 68.5 months, 65.3% male). Increased head circumference was noted in 40.8% of patients, and 24.4% reported preoperative headaches. Primary surgical treatments included CSF diversion in 61.2% of cases (42.9% VPS, 18.4% endoscopic third ventriculostomy [ETV]), intradural foramen magnum decompression (FMD) in 24.5%, and extradural FMD in 10.2%. The success-to-complication ratio was most favorable for extradural FMD (100% success, 20% complication), followed by ETV (44% success, 0% complication), VPS (76.2% success, 47.6% complication), and intradural FMD (33% success, 8.3% complication). At discharge and at the latest follow-up, 76% and 69% of patients showed improved Chicago Chiari Outcome Scale scores, respectively. The primary treatment modality and the bulging of the lamina terminalis were significant potential risk factors for the failure rate after multivariate analysis.

**Conclusion:**

Our data reveals considerable heterogeneity in treatment approaches. Extradural FMD exhibited the most favorable success–to–complication ratio, while intradural FMD had the least favorable ratio. These findings provide some basis for informed discussion with families. Larger prospective studies are still needed to refine treatment strategies.

**Supplementary Information:**

The online version contains supplementary material available at 10.1007/s00381-025-07052-4.

## Introduction

Chiari I malformation, defined as the caudal displacement of the cerebellar tonsils below the level of the foramen magnum, was first described by Hans Chiari, an Austrian Pathologist, in 1891[1]. Since its first description, the association between Chiari I malformation (CIM) and hydrocephalus has been described, however, its pathophysiology remains controversial [2]. Hydrocephalus occurs in 7–10% of all CIM patients and they can further develop a syringomyelia either due to the CIM or the hydrocephalus [3, 4]. However, the pathophysiology of hydrocephalus in CIM has not been established yet. One theory assumes that the tonsillar descent obstructs the fourth ventricular outflow and the basal subarachnoid space, causing obstructive hydrocephalus. Hence, a decompression of the foramen magnum would be the surgical intervention of choice to relieve the obstruction. However, patients with syndromic craniosynostosis have a high incidence of a small posterior fossa with CIM, but only a fraction of them develop hydrocephalus—which questions this direct cause-relation theory [2, 5]. A second theory assumes that the increased intracranial pressure (ICP) due to hydrocephalus, causes a secondary CIM by “pushing the tonsils” down and into the foramen magnum, leading to a transient and reversible caudal translation of the tonsils, potentially aggravating an existing minor and asymptomatic CIM [2]. The second theory would support cerebrospinal fluid (CSF) diversion as a first surgical intervention, which could be either achieved with the insertion of a ventriculoperitoneal shunt (VPS) or an endoscopic third ventriculostomy (ETV), depending on the nature of the hydrocephalus and patient-specific factors [2, 6, 7]. Given that the pathophysiology of patients with CIM and hydrocephalus has not yet been fully elucidated, the optimal treatment of these children remains controversial and is very heterogeneous among treating centers [2, 8].

This multicenter retrospective study aimed to analyze the different management strategies of children presenting with a CIM and concomitant hydrocephalus with or without syringomyelia and their respective outcomes.

## Methods

### Data collection

We conducted a retrospective chart review from 2005 to 2021, including children (< 18 years of age) undergoing surgical treatment for CIM with concomitant hydrocephalus with or without syringomyelia. Patients presenting with CIM without hydrocephalus were excluded. Children were treated at ten tertiary care centers in Switzerland, Germany, the United Kingdom, Italy, and Israel. The local ethics committees and institutional review boards approved the study protocol and waived patients’ consent due to the study’s retrospective nature according to local regulations.

Data was collected from patients’ records and imaging data. Baseline characteristics included gender, age, pathology, radiological findings such as the presence of ventriculomegaly assessed using Evans index (≥ 0.3), increased head circumference, platybasia, basilar invagination, scoliosis, and syrinx, as well as comorbidities. Evans index was defined as the ratio of the maximal anterior horn diameter and the maximal diameter of the internal skull [9]. Increased head circumference was defined as a value deemed pathological by the treating physician. Further, data related to the surgeries, their outcomes, and morbidity were assessed. The surgeries were categorized into extradural foramen magnum decompression (FMD), intradural FMD (both types of FMD included a laminectomy of C1), VPS insertion, ETV, and other. We collected data on whether an expansile duraplasty was performed and/or the tonsils were shrunken or resected during intradural FMD. 

The primary outcome was the rate of revision surgery due to treatment failure. We further assessed the time to revision surgery, the type of revision surgery, and the number of revision surgeries needed due to a treatment failure. Treatment failure was defined as the need for an additional surgery due to unsatisfactory treatment of the hydrocephalus and/or CIM by the primary surgical intervention. For patients treated with a VPS, treatment failure was defined when an additional treatment modality was needed (e.g., ETV and FMD), while shunt dysfunction leading to a shunt revision was defined as a complication. Further, shunt infection or an over-drainage resulting in subdural hematomas requiring an evacuation was referred to as complication as well. For ETV, failure was defined when repeat ETV with a refined/adapted surgical strategy was performed or other treatment modalities (e.g., VPS and FMD) were required. If the ETV was repeated using the same surgical steps as during the primary surgery, it was defined as a complication. The same definition was used for FMD. Some patients underwent revision surgery for both failure and complications, which were analyzed separately. Secondary outcomes included complications and the rates of revision surgery due to complications. Complications were divided into intraoperative complications including the need for blood transfusion, vascular or forniceal injury, and unwanted brain tissue damage, and postoperative complications including CSF fistula/pseudomeningocele, signs of increased intracranial pressure (ICP) such as altered consciousness, headaches, and vomiting, stroke, and superficial and deep infection, defined as wound infections requiring only antibiotic treatment (superficial) or additionally requiring a wound washout (deep), as well as new neurological deficits. We assessed the Chicago Chiari Outcome Scale (CCOS) postoperatively and at follow-up whenever available, evaluating pain, neurology, functionality, and possible complications on a four-point Likert scale [10]. The CCOS was dichotomized into worse (CCOS 4–8), unchanged (CCOS 9–12), and improved (CCOS 13–16). Postoperative radiological parameters assessed included the presence of ventriculomegaly using the Evans index, and/or syringomyelia, and whether the tonsillar descent has improved. Readmission and its underlying reason, hospitalization time, and discharge location (home, rehabilitation, other) were collected. Potential factors influencing the success and complication rate of treatment were analyzed.

### Statistical analysis

We conducted descriptive statistics presenting continuous data with a mean [± SD] and ordinal data with a median [IQR]. A log-rank test for the time to the first revision surgery due to failure or complication, stratified by the primary treatment modality, was carried out and presented as Kaplan–Meier curves. For this analysis, surgeries performed less than two times throughout the cohort were excluded. We used the Fisher exact test or the Chi-square test for categorical data and the Mann–Whitney *U* test for continuous data for comparative analysis. Potential factors influencing the success rates of treatment and complications were analyzed in a univariate model, while significant variables were then inserted into a step-down multivariable regression model and analyzed. Given the low number of patients included, we did not conduct any subgroup analysis for patients with underlying syndromes. All statistical analyses were carried out using the R statistical software (R Foundation for Statistical Computing, Vienna, Austria, version 4.3, 2023) and SPSS (SPSS, IBM, Chicago, New York). A two-sided *p*-value of < 0.05 was considered statistically significant.

## Results

### Demographics and baseline characteristics

We included 49 patients with an average age at diagnosis of 68.47 months (± 71.44, range 0–247 months) and 32 (65.3%) males. Most patients (*n* = 44, 89.8%) suffered preoperative symptoms; 12 patients (24.4%) presented with headaches, of which half were CIM-typical (*n* = 6, 12.2%) headaches. Twenty patients (40.8%) presented with increased head circumference. In most patients, hydrocephalus (*n* = 38, 77.6%) was the main suspected cause of symptoms. All patients had an Evans index above 0.3, indicating hydrocephalus, with a mean Evans index of 0.4 ± 0.08 (range 0.3–0.62). In 17 patients (36.2%), the third ventricle floor was reported to bulge downwards, indicating increased pressure gradient and non-communicating hydrocephalus. Syringomyelia concomitant to CIM and hydrocephalus was seen in 29 patients (59.2%). The baseline characteristics of the cohort are summarized in Table 1.

**Table 1:** Demographic parameters of the included patients

**Parameter**		**Overall *n* (%)**
*n*		49
Gender (%)	Female	17 (34.7)
	Male	32 (65.3)
Age at diagnosis (mean (SD))		68.47 (71.44)
No preop symptoms (%)	Yes	5 (10.2)
	No	44 (89.8)
Preop Chiari typical headaches (%)	Yes	6 (12.2)
	No	43 (87.8)
Preop atypical headache (%)	Yes	6 (12.2)
	No	43 (87.8)
Preop CN deficits (%)	Yes	6 (12.2)
	No	43 (87.8)
Preop vomitus (%)	Yes	5 (10.2)
	No	44 (89.8)
Preop failure to thrive (%)	Yes	1 (2.0)
	No	48 (98.0)
Preop sleep apnea (%)	Yes	2 (4.1)
	No	47 (95.9)
Preop sensory deficits (%)	Yes	5 (10.2)
	No	44 (89.8)
Preop motor deficits (%)	Yes	5 (10.2)
	No	44 (89.8)
Preop gait disturbance (%)	Yes	4 (8.2)
	No	45 (91.8)
Preop increase HC (%)	Yes	20 (40.8)
	No	29 (59.2)
Preop full fontanelle (%)	Yes	5 (10.2)
	No	44 (89.8)
Preop sunsetting (%)	Yes	1 (2.0)
	No	48 (98.0)
Preop vertigo (%)	Yes	2 (4.1)
	No	47 (95.9)
Preop other symptoms (%)	Yes	20 (40.8)
	No	29 (59.2)
Main symptom caused by CIM (%)	Yes	1 (2.1)
Main symptom caused by hydrocephalus (%)	Yes	36 (73.5)
Main symptom caused by syrinx (%)	Yes	3 (6.1)
Main symptom caused by other or multiple factors (%)	Yes	8 (16.3)
No comorbidities (%)	Yes	11 (22.4)
	No	38 (77.6)
Ehlers–Danlos (%)	No	49 (100.0)
Crouzon (%)	Yes	3 (6.1)
	No	46 (93.9)
Pfeiffer (%)	Yes	1 (2.0)
	No	48 (98.0)
Achondroplasia (%)	Yes	1 (2.0)
	No	48 (98.0)
Obesity (%)	Yes	1 (2.0)
	No	48 (98.0)
Heart disease (%)	Yes	4 (8.2)
	No	45 (91.8)
Tethered cord (%)	Yes	1 (2.0)
	No	48 (98.0)
Downs syndrome (%)	No	49 (100.0)
VACTERL (%)	No	49 (100.0)
Other Comorbidities (%)	Yes	28 (57.1)
	No	21 (42.9)
Papilla edema (%)	No	29 (87.9)
	Yes	4 (12.1)
Evans Index preoperatively (mean (SD))		p = 0.85
Overall		4.0 (0.08)
VP-shunt group		0.40 (0.08)
ETV		0.39 (0.05)
Extradural FMD		0.37 (0.06)
Intradural FMD		0.39 (0.07)

CSF diversion was the primary surgical treatment modality in 30 patients (61.2%). Twenty-one patients (42.9%) were initially treated by VPS, and nine patients (18.4%) underwent an ETV. Twelve patients (24.5%) underwent intradural FMD, including expansile duraplasty and C1 laminectomy, and five (10.2%) were treated with extradural FMD and C1 laminectomy (Table 2). For the intradural FMD, five patients (10.2%) had additional tonsillar shrinking or resection, and in six patients (12.2%), arachnoid adhesions were dissected (Table 2). Two patients (4.2%) were allocated to “other surgeries,” including one fatty filum untethering and one cranial vault expansion due to complex craniosynostosis. The Evans index did not seem to influence decision-making concerning the primary surgical treatment modality since the mean Evans index between the groups was comparable (Table 1). The mean follow-up time of the cohort was 49.5 (± 54.0) months.

**Table 2:** Primary surgery including technical details

**Parameters**		**Overall n (%)**
*n*		49
Time to primary surgery since diagnosis (mean (SD)), months		11.76 (23.48)
Primary surgery (%)	Cranial vault reconstruction	1 (2.1)
	ETV	9 (18.4)
	Filum untethering	1 (2.1)
	FMD extradural	5 (10.2)
	FMD intradural	12 (24.5)
	VP-shunt	21 (42.9)
FMD extradural (%)	Bony decompression only	3 (60)
	Bony decompression with dural splitting	1 (20)
	Not further described	1 (20)
FMD intradural (extraarachnoidal) (%)	Yes	1 (8,3)
	No	11 (91.7)
FMD intradural (opening of arachnoidea and webs) (%)	Yes	6 (50)
	No	6 (50)
FMD intradural (tonsillar shrinking) (%)	Yes	4 (33.3)
	No	8 (66.7)
FMD intradural (tonsillar resection) (%)	Yes	1 (8.3)
	No	11 (91.6)
Duraplasty periosteal graft (%)	No	12(100.0)
Duraplasty bovine graft (%)	No	12(100.0)
Duraplasty artificial graft (%)	Yes	4 (33.3)
	No	8 (66.7)
Duraplasty other (%)	Yes	2 (16.6)
	No	10 (83.4)

### Revision surgery due to failure of primary treatment modality

**Fig. 1:**
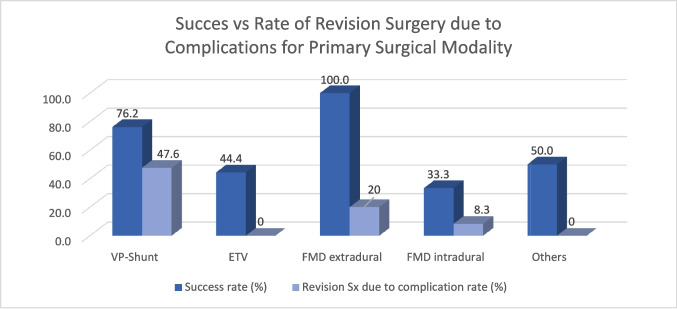
Success and revision rate due to complications stratified by the primary treatment modality

Overall, in 19 patients (38.8%), the primary treatment modality failed, leading to subsequent surgeries. Intradural FMD had the highest failure rate (*n* = 8, 66.7%), while patients with an extradural FMD had no failures (Table 3, Fig. 1). Patients treated with an ETV had a failure rate of 55.6% (*n* = 5), compared to 23.8% (*n* = 5) for patients treated with a VPS. A significant difference between the type of primary treatment and the failure rate was observed (Supplementary Table 1, *p* = 0.03). The univariate analysis further identified a preoperative deviation of the lamina terminalis (Supplementary Table 1, *p* = 0.02) and the study center (Supplementary Table 1, *p* = 0.002) as factors significantly influencing the failure rate.

**Table 3:** Patients undergoing revision surgery due to failure of the primary treatment

Center	Gender	Age (m)	Cause of main symptom	Primary treatment	Secondary treatment	Tertiary treatment	Quaternary treatment
#7	Male	6	Other/multiple	Cranial vault expansion	VPS insertion		
#5	Male	121	Hydrocephalus	ETV	Repeat ETV	FMD intradural	
#8	Female	79	Hydrocephalus	ETV	FMD intradural		
#8	Female	91	Hydrocephalus	ETV	VPS insertion		
#8	Male	16	Hydrocephalus	ETV	VPS insertion (after EVD/ICP)		
#10	Female	95	Other/multiple	ETV	Syringosubarachnoid shunt		
#2	male	24	Hydrocephalus	FMD intradural	ETV	Filum untethering	VPS insertion
#4	Female	141	Syrinx	FMD intradural	FMD intradural		
#5	Female	17	Other/multiple	FMD intradural	VPS insertion		
#5	Male	41	Hydrocephalus	FMD intradural	VPS insertion		
#5	Male	21	Hydrocephalus	FMD intradural	ETV (after EVD/ICP)		
#7	Male	208	Other/multiple	FMD intradural	FMD intradural		
#7	Male	122	Other/multiple	FMD intradural	VPS insertion (after EVD/ICP)		
#9	Male	12	Other/multiple	FMD intradural	VPS insertion		
#1	Male	8	Hydrocephalus	VPS	FMD extradural		
#2	Male	0	Hydrocephalus	VPS	FMD intradural	Re-FMD and occipital expansion	
#10	Female	8	Hydrocephalus	VPS	FMD intradural		
#10	Male	6	Hydrocephalus	VPS	Syringosubarachnoid shunt	FMD intradural	
#10	Female	111	Hydrocephalus	VPS	FMD intradural	Syringosubarachnoid shunt

After multivariable analysis, the primary treatment modality and a deviation of the lamina terminalis remained potential risk factors significantly influencing the failure rate (*p* = 0.047 and *p* = 0.004, Supplementary Table 1).

### Revision surgery due to complications

Overall, 18 patients (36.7%) underwent 27 revision surgeries due to complications (Table 4, Fig. 1). VPS insertion showed the highest complication rate (*n* = 10, 47.6%), followed by ETV (*n* = 2, 22.4%), extradural (*n* = 1, 20%), and intradural FMD (*n* = 1, 8.3%). In six patients (30%), complications occurred during their secondary or tertiary treatment (Table 4). Evans index and age at diagnosis significantly influenced the complication rate in univariate analysis, while after multivariable analysis, only preoperative Evans index remained significantly associated with higher complication rates (Supplementary Table 2).

**Table 4:** Patients undergoing revision surgery due to postoperative complications

Center	Gender	Age (m)	Cause of main symptom	Primary treatment	Complications	Treatments
#1	Male	7	Hydrocephalus	VPS (primary)	1.CSDH 2. CSDH 3. CSF fistula	1.BLT/VPS removal 2. BLT 3. VPS
#1	Male	247	Other/multiple	FMD extradural	1.Wound infection	1.Surgical washout
#2	Male	0	Hydrocephalus	VPS (primary)	1.VPS dysfunction 2. VPS dysfunction 3. VPS dysfunction 4. VPS infection	1.VPS revision 2. VPS revision 3. VPS revision 4. VPS externalization
#4	Male	15	Hydrocephalus	FMD intradural	1.Wound infection	1.Surgical washout
#6	Male	58	Other/multiple	VPS (primary)	1.VPS infection	1.VPS externalization
#6	Male	106	Hydrocephalus	VPS (primary)	1.other	1.VPS externalization/internalization
#6	Male	102	Hydrocephalus	VPS (primary)	1.VPS dysfunction 2. VPS dysfunction 3. VPS dysfunction 4. VPS distal dislocation 5. VPS distal dislocation	1.VPS revision 2. VPS externalization/internalization 3. VPS revision 4. VPS revision 5. VPS revision
#7	Male	6	Other/multiple	VPS (after failed Cranial vault expansion)	1.VPS dysfunction	1.VPS revision
#7	Male	122	Other/multiple	VPS (after failed FMD intradural)	1.VPS dysfunction	1.VPS revision
#8	Female	91	Hydrocephalus	VPS (after failed ETV)	1.VPS dysfunction	1.VPS revision
#8	Male	16	Hydrocephalus	VPS (after failed ETV)	1.VPS dysfunction	1.VPS revision
#9	Female	2	Hydrocephalus	VPS (primary)	1.VPS overdrainage	1.VPS revision
#9	Male	7	Hydrocephalus	VPS (primary)	1.VPS infection	1.distal VPS explantation/VAS
#9	Male	13	Hydrocephalus	VPS (after failed FMD intradural)	1.VPS dysfunction	1.VPS revision
#9	Male	12	Other/multiple	VPS (primary)	1.VPS dysfunction	1.VPS revision
#10	Female	8	Hydrocephalus	VPS (primary)	1.CSDH	1.BLT
#10	male	6	Hydrocephalus	SSS (after failed VPS)	1.CSF fistula	1.thoracic wound revision
#10	Female	111	Hydrocephalus	VPS (primary)	1.VPS dysfunction 2. VPS infection	1.VPS revision 2. VPS externalization/antibiotics/internalization

### Comparison of success rate versus complication rate of primary surgery

The success versus complication resulting in a need for revision surgery rate was most favorable for extradural FMD (100% success rate, 20% complication rate), followed by ETV (44% success rate, 0% complication rate), VPS (76.2% success rate, 47.6% complication rate), and intradural FMD (33% success rate, 8.6% complication rate, Fig. 1). Intradural FMD showed significantly higher failure rates than any other primary surgical method (*p* = 0.03, Supplementary Table 1).

## Clinical outcome and postoperative follow-up

**Fig. 2:**
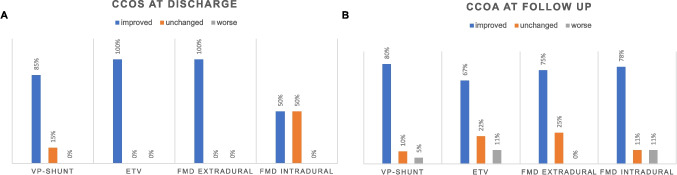
CCOS at discharge and follow-up for the primary treatment modalities

The outcome at discharge based on the CCOS improved in 37 patients (76%) and remained unchanged in 9 patients (18%). No patient showed worsening of the symptoms (Supplementary Table 3, Fig. 2A). Outcomes at discharge differed significantly among the primary treatment modalities, with intradural FMD showing significantly less improved outcomes at discharge than the other treatment modalities (Fig. 2A). At the most recent follow-up, three patients (6.1%) described a worse outcome and seven patients (14%) showed unchanged outcomes, while 34 patients (69%) showed improved outcomes. No significant difference between the primary treatment modalities was observed at this time point (Supplementary Table 3, Fig. 2B).

Sixteen patients were readmitted (32.7%), of which six (37.5%) were elective re-admissions, eight patients (16.8%) showed worsening symptoms or new neurologic deficits, and two (2.2%) had other reasons for a re-admission.

## Differences in baseline and outcome stratified by the primary treatment modalities

**Fig. 3:**
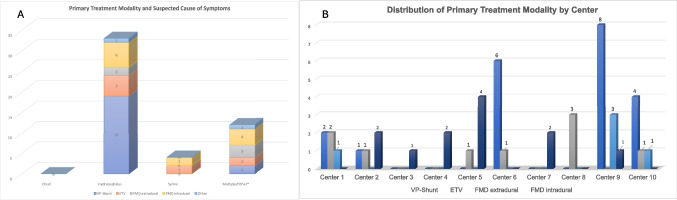
**A** Primary treatment modality according to the suspected cause of symptoms of these, (* six patients with Chiari typical symptoms). **B** Distribution of primary treatment modality according to the different study centers

Patients with a VPS or “other modality” as their primary surgery were significantly younger compared to the remaining children (*p* = 0.015, Table 5). The rate of CIM or syringomyelia being the main cause of symptoms was not significantly different between the treatment modalities; however, in patients with hydrocephalus as the leading cause of their symptoms, significantly more VPS insertions or “other” treatment modalities were observed (*p* = 0.01, Fig. 3A). Patients undergoing intradural FMD had a significantly longer hospital stay than the other treatment groups (*p* = 0.05). Two patients were transferred to another hospital or an inpatient rehabilitation facility, while the remaining patients were all discharged home (*p* = 0.02, Table 5).

**Table 5:** Outcome parameters stratified by the primary treatment modalities

	**ETV**	**FMD extradural**	**FMD intradural**	**Other**	**VP-shunt**	***p*-value**
*n*	9	5	12	2	21	
**Intraoperative complications (%)**	0 (0.0)	0 (0.0)	0 (0.0)	0 (0.0)	1 (4.8)	0.851
**Hospitalization time [days] (mean (SD))**	3.33 (2.40)	8.60 (9.79)	11.88 (6.40)	5.50 (3.54)	6.16 (5.34)	**0.05**
**Discharge location (%)**						**0.022**
Home	9 (100.0)	3 (60.0)	11 (100.0)	2 (100.0)	21 (100.0)	
Other hospital/clinic	0 (0.0)	1 (20.0)	0 (0.0)	0 (0.0)	0 (0.0)	
Rehabilitation	0 (0.0)	1 (20.0)	0 (0.0)	0 (0.0)	0 (0.0)	
**Symptoms at discharge (%)**						0.491
Improvement of symptoms	4 (44.4)	2 (40.0)	3 (30.0)	0 (0.0)	9 (45.0)	
No symptoms	4 (44.4)	1 (20.0)	2 (20.0)	1 (50.0)	8 (40.0)	
Unchanged	1 (11.1)	2 (40.0)	5 (50.0)	1 (50.0)	3 (15.0)	
**Pain at discharge (%)**						**0.001**
Improved or controlled with medication	1 (14.3)	2 (50.0)	5 (83.3)	1 (50.0)	5 (27.8)	
Resolved	6 (85.7)	2 (50.0)	1 (16.7)	0 (0.0)	13 (72.2)	
Unchanged and refractory to medication	0 (0.0)	0 (0.0)	0 (0.0)	1 (50.0)	0 (0.0)	
**Chicago Outcome Score (mean (SD))**	15.50 (0.84)	14.50 (1.29)	12.67 (1.51)	12.00 (2.83)	14.50 (1.69)	**0.015**

Patients after VPS insertion had the highest rate of resolved pain postoperatively, which was significantly higher compared to the other patients (72.2%, *p* = 0.001, Table 5). Further, patients after ETV had a significantly higher total CCOS, while patients having “other” surgeries scored lowest (*p* = 0.015, Table 5, Supplemental Table 1).

### Treatment in the different centers

The centers differed in some of the patients’ baseline parameters (Supplementary Table 4). Some centers had patients (centers 7 and 8) whose symptoms were mainly caused by CIM, while for other centers, hydrocephalus was the main cause of symptoms (centers 2 and 9).

The initial treatment varied significantly among the different centers (*p* = 0.003, Fig. 3B, Supp Table 2). Three centers (centers 6, 8, and 9) mainly performed a CSF diversion as first-line therapy (either an ETV or VPS), while center 9 performed a VPS in most cases (> 60%). Meanwhile, other centers (centers 3, 4, 5, and 7) performed an intradural FMD in most of their cases. Other centers (centers 1 and 10) had a more heterogeneous distribution of their primary surgery (Fig. 3B). No clear correlation was observed between the main suspected cause of symptoms and the choice of primary treatment modality, either in the overall cohort or in any of the individual centers.

## Discussion

This multicenter retrospective cohort study investigated the success and failure rate of multiple treatment options in children with CIM and associated hydrocephalus with or without syringomyelia. We included 49 children from 10 centers. This trial shows that treatment algorithms for these patients are very heterogeneous, while no standard of care for CIM hydrocephalus patients could be identified. Whether the hydrocephalus or the CIM was treated first did not significantly impact re-operation rates and outcomes. The most common primary treatment modality was the insertion of a VPS (*n* = 21, 42.9%), followed by intradural FMD (*n* = 12, 24.5%). Overall, 28 (57.1%) patients underwent 63 additional surgeries due to treatment failure or complications. Extradural FMD showed the lowest rate (20%) of repeat surgery (due to failure or complications), followed by ETV (60%), VPS (70%), and intradural FMD (80%). Multivariate analysis showed that a deviation of the lamina terminalis on preoperative MRI and primary treatment modality (intradural FMD) was associated with higher failure rates, while a higher Evans index on preoperative MRI was significantly associated with complications. At the most recent follow-up, 69% of the patients showed improved outcomes based on the CCOS.

Despite a large body of evidence on the treatment for CIM, the pathophysiology of CIM remains ambiguous, and therefore the optimal treatment remains controversial [2, 4, 6–8, 11, 12]. The most recent guidelines from the American Association of Neurological Surgery state that patients with a CIM, regardless of their syrinx status, can either be treated with an intra- or extradural FMD [13]. However, these guidelines do not include patients with hydrocephalus, where the dilemma of “what came first - the chicken or the egg” remains: Is the hydrocephalus caused by a narrowing of the foramen magnum due to the CIM or is the CIM caused by the hydrocephalus pushing the tonsils downwards? The heterogeneous distribution of the primary treatment modality within our cohort, including multiple international centers, clearly reflects this dilemma. While none of the patients with extradural FMD required additional surgery due to treatment failure, patients undergoing intradural FMD showed failure in one-third of the patients. In contrast to prior studies, extradural FMD showed in our cohort higher complication rates (20%, *n* = 1) than intradural FMD (8.3%, *n* = 1) [14]. This is most probably attributed to the rather small patient groups of each treatment modality, since in both groups, one complication occurred, namely a postoperative wound infection.

Assuming the hydrocephalus is the cause of CIM by pushing down the tonsils, the primary treatment is the insertion of a VPS or performing an ETV aiming to resolve the hydrocephalus. In our cohort, most patients were primarily treated with a CSF diversion surgery (VPS: *n* = 21, 42.8%; ETV: *n* = 9, 18.4%), most probably, since in most of the patients (73.5%) within our cohort, the main symptoms were attributed to hydrocephalus and not CIM. This is in line with previous reports showing that the majority of neurosurgeons tend to treat the hydrocephalus first [11]. Although VPS had a relatively high success rate (76.2%), suggesting that treating primarily the hydrocephalus seems justified, VPS led also to the highest rate of revision surgery due to complications, among all treatment modalities. ETV, which has been presented in recent reports as a viable option to treat CIM concomitant with hydrocephalus with or without the presence of syringomyelia [11, 12], might be a valid alternative to VPS leading to fewer revision surgeries due to complications. In the present cohort, ETV led to a success rate of 40%, with none of the patients undergoing revision surgery due to complications. Overcrowding of the posterior fossa was identified as a factor potentially impairing the success of ETV [12]. We did not assess the volume of the posterior fossa in our cohort; however, all other assessed factors (age, deviation of lamina terminalis of floor of the 3rd ventricle, main symptom at presentation, Evans index, time to surgery, basilar invagination, platybasia) showed no correlation with failure of ETV (results not shown). A series by Hayhurst et al. investigated 16 children with CIM and hydrocephalus undergoing ETV as their primary treatment, of which all but one remained shunt-free. However, nearly 40% required an additional FMD [15]. Similar results were observed in other small case series [16, 17]. In our series, two patients (22.2%) initially treated with ETV eventually underwent intradural FMD, while two required (22.2%) a VPS, and one patient (11.1%) underwent SSS (Table 3). Based on the available literature and our results, drawing a definite conclusion on whether ETV or VPS should be preferred in patients with CIM and hydrocephalus is impossible. 

Although in our cohort, in most patients, the symptoms were not primarily attributed to the CIM, 17 patients (34.7%) underwent FMD (intradural FMD: *n* = 12, 24.5%; extradural FMD: *n* = 5, 10.2%) as their primary treatment. However, in about one-third of the patients (*n* = 5), an additional CSF diversion procedure (4 patients (80%) underwent a VPS, and 1 patient (20%) underwent an ETV) was required. Only patients undergoing intradural FMD required subsequent surgery. This could be explained by the fact that intradural surgery could worsen hydrocephalus due to possible arachnoid scarring or changed pressure relations after the decompression 18, 19]. Previous case series showed that most patients who underwent revision surgery after intradural FMD presented with severe arachnoid scarring^18^. Based on our findings, it seems that extradural FMD within the context of CIM and hydrocephalus might be superior to intradural FMD.

C1–C2 fixation has been advocated by some authors for the treatment of CIM [20–22]. They hypothesize that CIM is caused by a C1–C2 instability, either subtle or overt, and the observed tonsillar herniation is a reaction of the body to this instability and serves as protection for the spinal cord [20, 21]. In our cohort, none of the patients underwent a C1–C2 fixation, which remains a last-resort treatment modality for CIM in most centers [23]. However, given that odontoid invagination may develop later in some patients, particularly those with a syndromic background, long-term follow-up into adulthood is recommended. Additionally, patients with craniofacial syndromes or achondroplasia exhibit a higher rate of hydrocephalus than other patients with CIM [5, 24]. The proposed mechanism involves venous hypertension resulting from jugular foramen stenosis, leading to increased intracranial pressure and hydrocephalus [24, 25]. Our cohort included four patients with underlying syndromes, contributing to the heterogeneity of the included patients. However, this diversity accurately reflects the real-life cohort of patients presenting with CIM and concomitant hydrocephalus. The possibly different pathophysiology needs to be accounted for when making treatment decisions, and one of these patients also underwent cranial vault expansion as a treatment, highlighting the need for individualized treatment approaches.

In cases of CIM concomitant with hydrocephalus and syringomyelia, an additional dilemma arises concerning the pathophysiology of the syringomyelia, namely, is it due to the CIM, the hydrocephalus, or a combination of both. Since syringomyelia occurs within the context of CIM but also hydrocephalus, this issue remains unclear. When comparing patients presenting with syringomyelia (*n* = 29) and without syringomyelia within our cohort, no difference was seen in terms of leading symptoms, primary treatment modality chosen, failure or complication rate, and clinical outcome. To note, all patients treated with ETV presented with additional syringomyelia, as well as more often with a basilar invagination and scoliosis on preoperative imaging. Further, overall, 60% of the patients presenting with syringomyelia underwent a CSF diversion procedure (ETV or VPS), while none of these patients underwent SSS as the primary treatment modality. In two cases showing progressive syringomyelia after initial treatment, SSS was completed, in one case after initial ETV and in the other case after VPS insertion. The treatment of syringomyelia remains controversial, as some advocate the placement of a syringo-subarachnoid, peritoneal, or pleural shunt [26, 27], while others recommend the placement of a 4th ventricular stent [28].

The descriptive findings of our series suggest that the theory that the caudal migration of the tonsils causes intermittent obstructive hydrocephalus, which can be resolved by removing the crowding at the cranio-cervical junction (CCJ), is applicable. Concomitantly, the theory that HCP causes tonsillar caudal migration leading to CIM might be applicable as well [2]. It seems plausible that both mechanisms play a simultaneous role in the formation of CIM and hydrocephalus. Further, it seems that the main symptom is not necessarily suggestive of the pathophysiological mechanism leading to CIM and hydrocephalus and might be misleading when it comes to deciding which primary surgical modality should be applied. Prospective, multicenter trials meticulously evaluating the patients’ symptoms at presentation and comparing the different treatment modalities, while putting them into context with the patients’ main symptoms, imaging features, and finally their outcome, are needed to potentially reach firm conclusions on the ideal treatment of CIM and hydrocephalus patients.

## Limitations

This retrospective cohort study is subject to all the limitations of data collection inherent in such work. The data was collected in ten different centers, and since no clear guidelines exist for the treatment of concomitant CIM and hydrocephalus, the applied treatment was heterogeneous among the centers and, at times, even within the centers. This is clearly reflected in the significant difference in the primary treatment modality applied among the different centers. This is amplified by the fact that the centers recruited variable numbers of patients, which can skew the statistical analysis, and the study is not powered for a thorough analysis across the different centers. The follow-up time and interval differed among centers, with some patients being followed multiple times while others only had one follow-up visit registered, leading to a potential bias. The interval of radiographic follow-ups varied among centers, and not many radiographic outcomes were available during follow-up. Moreover, we included patients with and without syringomyelia, which leads to a more heterogeneous patient group, adding more variables to the equation and potentially skewing our results. A selection bias cannot be excluded, as some patients presenting with hydrocephalus accompanied by concomitant CIM might have been diagnosed solely with hydrocephalus, without recognition or documentation of the associated CIM. Consequently, these patients were not registered as CIM cases.

This multicenter cohort study, which investigates various treatment modalities and their outcomes in 49 children with CIM and concomitant hydrocephalus, is, to our knowledge, the largest of its kind to date. Encompassing data from ten experienced pediatric neurosurgical centers across Europe, it offers valuable insights into the realities of daily clinical practice and contributes valuable outcome data for this rare and complex condition in children.

## Conclusion

The management of children presenting with concurrent CIM and hydrocephalus remains a complex challenge. Our data reveals considerable heterogeneity in treatment approaches both among and within different medical centers. While 76% of cases achieved favorable clinical outcomes measured by the CCOS, approximately one-third of patients required multiple surgical interventions. Extradural FMD demonstrated a favorable success-to-complication ratio, whereas intradural FMD exhibited the least favorable ratio. ETV and VPS remain feasible treatment modalities to address hydrocephalus within the context of CIM.

These findings provide a framework for informed discussions with families navigating these intricate conditions. However, larger prospective studies are still required to refine treatment strategies and to create evidence-based guidelines.

## Supplementary Information

Below is the link to the electronic supplementary material.

ESM 1 DOCX (59.3 K)

## Data Availability

No datasets were generated or analysed during the current study.

## Declarations

### Previous presentations

European Society for Pediatric Neurosurgery, Abstract presentation, London, UK, May 2025.
