# Supplementary material for: The management of pediatric Chiari I malformation with concomitant hydrocephalus: a multicenter retrospective analysis
Source: Childs Nerv Syst. 2025 Nov 25;41(1):378. doi: 10.1007/s00381-025-07052-4 (PMC12644218; doi:10.1007/s00381-025-07052-4)
Supplement: Supplementary file 1 — DOCX (59.3 K) [file 381_2025_7052_MOESM1_ESM.docx]

**Supplementary Tables**

**Supplementary Table 1:** Uni- and multivariable analysis of potential factors influencing failure rate of primary treatments

| **Univariate Analysis** | | | |  | **Multivariable Analysis** | | | |
| --- | --- | --- | --- | --- | --- | --- | --- | --- |
| **Parameters** |  | | **p-value** |  | **Exp(B)** | **SE** | **95% CI** | **p-value** |
|  | **Failure** | |  |  |  |  |  |  |
|  |  |  |  |  |  |  |  |  |
|  | **Yes** | **No** |  |  |  |  |  |  |
| **Primary surgery** |  |  | **p=0.03** |  | 1.767 | 0.286 | 1.008 -3.094 | **0.047** |
| VP-Shunt | 5 (23.8%) | 16 (76.2%) |  |  |  |  |  |  |
| ETV | 5 (55.6%) | 4 (44.4%) |  |  |  |  |  |  |
| FMD extradural | 0 (0%) | 5 (100%) |  |  |  |  |  |  |
| FMD intradural | 8 (66.7%) | 4 (33.3%) | * |  |  |  |  |  |
| Others | 1 (50%) | 1 (50%) |  |  |  |  |  |  |
|  |  |  |  |  |  |  |  |  |
| **Leading Symptom** |  |  | p=0.61 |  |  |  |  |  |
| Hydrocephalus | 13 (36.1%) | 23 (63.9%) |  |  |  |  |  |  |
| Syringomyelia | 1 (33.3%) | 2 (66.7%) |  |  |  |  |  |  |
| Chiari | 0 (0%) | 1 (100%) |  |  |  |  |  |  |
| Other/multiple* | 5 (55.6%) | 4 (44.4%) |  |  |  |  |  |  |
|  |  |  |  |  |  |  |  |  |
| **Basilar invagination** |  |  | p=0.17 |  |  |  |  |  |
| yes | 3 (75%) | 16 (37.2%) |  |  |  |  |  |  |
| no | 1 (25%) | 27 (62.8%) |  |  |  |  |  |  |
| unknown | 0 | 2 |  |  |  |  |  |  |
|  |  |  |  |  |  |  |  |  |
| **Platybasia** |  |  | p=0.66 |  |  |  |  |  |
| yes | 3 (15.8%) | 6 (20.7%) |  |  |  |  |  |  |
| no | 16 (84.2%) | 23 (79.3%) |  |  |  |  |  |  |
| unkown | 0 | 1 |  |  |  |  |  |  |
|  |  |  |  |  |  |  |  |  |
| **Scoliosis** |  |  | p=0.43 |  |  |  |  |  |
| yes | 5 (55.6%) | 10 (40%) |  |  |  |  |  |  |
| no | 4 (44.4%) | 15 (60%) |  |  |  |  |  |  |
| unkown | 4 | 10 |  |  |  |  |  |  |
|  |  |  |  |  |  |  |  |  |
| **Syrinx** |  |  | p=0.25 |  |  |  |  |  |
| yes | 14 (77.8%) | 15 (57.7%) |  |  |  |  |  |  |
| no | 4 (22.2%) | 11 (42.3%) |  |  |  |  |  |  |
| unkown | 1 | 4 |  |  |  |  |  |  |
|  |  |  |  |  |  |  |  |  |
| **Papilledema** |  |  | p=0.10 |  |  |  |  |  |
| yes | 1 (6.3%) | 14 (82.4%) |  |  |  |  |  |  |
| no | 15 (93.7%) | 3 (17.6%) |  |  |  |  |  |  |
| unkown | 3 | 12 |  |  |  |  |  |  |
|  |  |  |  |  |  |  |  |  |
| **Deviation of lamina terminalis** |  |  | **p=0.02** |  | 0.123 | 0.731 | 0.029 - 0.515 | **0.004** |
| yes | 11 (57.9%) | 6 (24%) |  |  |  |  |  |  |
| no | 8 (42.1%) | 19 (76%) |  |  |  |  |  |  |
| unkown | 0 | 4 |  |  |  |  |  |  |
|  |  |  |  |  |  |  |  |  |
| **Center** |  |  | **p=0.002** |  | 1.254 | 0.129 | 0.974 – 1.615 | 0.079 |
| #1 | 1 (20%) | 4 (80%) |  |  |  |  |  |  |
| #2 | 2 (40%) | 3 (60%) |  |  |  |  |  |  |
| #3 | 0 | 1 (100%) |  |  |  |  |  |  |
| #4 | 1 (50%) | 1 (50%) |  |  |  |  |  |  |
| #5 | 4 (80%) | 1 (20%) | * |  |  |  |  |  |
| #6 | 0 | 1 (100%) |  |  |  |  |  |  |
| #7 | 3 (30%) | 7 (70%) | * |  |  |  |  |  |
| #8 | 3 (100%) | 0 | * |  |  |  |  |  |
| #9 | 1 (8.3%) | 11 (91.7) | * |  |  |  |  |  |
| #10 | 4 (66.7%) | 2 (33.3%) |  |  |  |  |  |  |
|  |  |  |  |  |  |  |  |  |
| **Age (m)** | 59.3 (60.2) | 74.3 (78.1) | p=0.76 |  |  |  |  |  |
|  |  |  |  |  |  |  |  |  |
| **Evans Index preoperatively** | 0.40 (0.09) | 0.39 (0.07) | p=0.53 |  |  |  |  |  |
|  |  |  |  |  |  |  |  |  |

*Significant difference to the rest of the parameters within the group, SE = standard error

**Supplementary Table 2:** Uni- and multivariable analysis of potential factors influencing complication rate

| **Univariate Analysis** | | | |  | **Multivariable Analysis** | | | |
| --- | --- | --- | --- | --- | --- | --- | --- | --- |
| **Parameters** |  |  | **p-value** |  | **Exp(B)** | **SE** | **95% CI** | **p-value** |
|  | **Complications** | |  |  |  |  |  |  |
|  | yes | no |  |  |  |  |  |  |
| **Primary surgery** |  |  |  |  |  |  |  |  |
| VP-Shunt | 10 (47.6%) | 11 (54.2%) | * |  |  |  |  |  |
| ETV | 0 (0%) | 9 (100%) |  |  |  |  |  |  |
| FMD extradural | 1 (20%) | 4(80%) |  |  |  |  |  |  |
| FMD intradural | 1 (8.3%) | 11 (91.7%) |  |  |  |  |  |  |
| Filum untethering | 0 (0%) | 1 (100%) |  |  |  |  |  |  |
| Cranial vault expansion | 0 (0%) | 1 (100%) |  |  |  |  |  |  |
|  |  |  |  |  |  |  |  |  |
| **Additional surgery due to failure** |  |  | p=0.08 |  |  |  |  |  |
| yes | 9 (47.4%) | 10 (52.6%) |  |  |  |  |  |  |
| no | 7 (23.3%) | 23 (76.7%) |  |  |  |  |  |  |
|  |  |  |  |  |  |  |  |  |
| **Pathology causing main symptoms** |  |  | p=0.39 |  |  |  |  |  |
| Chiari | 0 | 1 (100%) |  |  |  |  |  |  |
| Hydro | 14 (38.9%) | 22 (61.1%) |  |  |  |  |  |  |
| Syrinx | 0 | 3 (100%) |  |  |  |  |  |  |
| Other | 2 (22.2%) | 7 (77.8%) |  |  |  |  |  |  |
|  |  |  |  |  |  |  |  |  |
| **Preoperative Evan’s Index (±SD)** | 0.45 (0.09) | 0.37 (0.05) | **p=0.01** |  | 3.28 | 5.788 | 1.734 – 4.826 | **.004** |
|  |  |  |  |  |  |  |  |  |
| **Comorbidities** |  |  | p=0.67 |  |  |  |  |  |
| no | 13 (34.2%) | 25 (65.8%) |  |  |  |  |  |  |
| yes | 3 (27.3%) | 8 (72.7%) |  |  |  |  |  |  |
|  |  |  |  |  |  |  |  |  |
| **Ehler Danlos** |  |  |  |  |  |  |  |  |
| no | 16 (32.7%) | 33 (67.3%) |  |  |  |  |  |  |
| yes | 0 | 0 |  |  |  |  |  |  |
|  |  |  |  |  |  |  |  |  |
| **Crouzon** |  |  | p=0.98 |  |  |  |  |  |
| no | 15 (32.6%) | 31(67.4%) |  |  |  |  |  |  |
| yes | 1 (33.3%) | 2 (66.7%) |  |  |  |  |  |  |
|  |  |  |  |  |  |  |  |  |
| **Pfeiffer** |  |  | p=0.48 |  |  |  |  |  |
| no | 16 (33.3%) | 32 (66.7%) |  |  |  |  |  |  |
| yes | 0 | 1 (100%) |  |  |  |  |  |  |
|  |  |  |  |  |  |  |  |  |
| **Achondroplasia** |  |  | p=0.18 |  |  |  |  |  |
| no | 15 (31.2%) | 33 (68.8%) |  |  |  |  |  |  |
| yes | 1 (100%) | 0 |  |  |  |  |  |  |
|  |  |  |  |  |  |  |  |  |
| **Obesity** |  |  | p=0.48 |  |  |  |  |  |
| no | 16 (33.3%) | 32 (66.7%) |  |  |  |  |  |  |
| yes | 0 | 1 (100%) |  |  |  |  |  |  |
|  |  |  |  |  |  |  |  |  |
| **Heart disease** |  |  | p=0.73 |  |  |  |  |  |
| no | 15 (33.3%) | 30 (66.7%) |  |  |  |  |  |  |
| yes | 1 (25%) | 3 (75%) |  |  |  |  |  |  |
|  |  |  |  |  |  |  |  |  |
| **Tethered cord** |  |  | p=0.48 |  |  |  |  |  |
| no | 16 (33.3%) | 32 (66.7%) |  |  |  |  |  |  |
| yes | 0 | 1 (100%) |  |  |  |  |  |  |
|  |  |  |  |  |  |  |  |  |
| **Down Syndrome** |  |  |  |  |  |  |  |  |
| no | 16 (32.7%) | 33 (67.3%) |  |  |  |  |  |  |
| yes | 0 | 0 |  |  |  |  |  |  |
|  |  |  |  |  |  |  |  |  |
| **VACTERL** |  |  |  |  |  |  |  |  |
| no | 16 (32.7%) | 33 (67.3%) |  |  |  |  |  |  |
| yes | 0 | 0 |  |  |  |  |  |  |
|  |  |  |  |  |  |  |  |  |
| **Other comorbidity** |  |  | p=0.93 |  |  |  |  |  |
| no | 7 (33.3%) | 14 (66.7%) |  |  |  |  |  |  |
| yes | 9 (32.1%) | 19 (67.9%) |  |  |  |  |  |  |
|  |  |  |  |  |  |  |  |  |
| **Center** |  |  | p=0.45 |  |  |  |  |  |
| #1 | 1 (20.0%) | 4 (80.0%) |  |  |  |  |  |  |
| #1 | 1(20.0%) | 4 (80.0%) |  |  |  |  |  |  |
| #2 | 1(20.0%) | 4 (80.0%) |  |  |  |  |  |  |
| #3 | 0 | 1 (100%) |  |  |  |  |  |  |
| #4 | 0 | 2 (100%) |  |  |  |  |  |  |
| #5 | 0 | 5 (100%) |  |  |  |  |  |  |
| #6 | 3 (42.9%) | 4 (57.1%) |  |  |  |  |  |  |
| #7 | 2 (66.7%) | 1 (33.3%) |  |  |  |  |  |  |
| #8 | 2 (66.7%) | 1 (33.3%) |  |  |  |  |  |  |
| #9 | 4 (33.3%) | 8 (66.7%) |  |  |  |  |  |  |
| #10 | 3 (50.0%) | 3 (50.0%) |  |  |  |  |  |  |
|  |  |  |  |  |  |  |  |  |
| **Gender** |  |  | p=0.32 |  |  |  |  |  |
| male | 12 (37.5%) | 20 (62.5%) |  |  |  |  |  |  |
| female | 4 (23.5%) | 13 (76.5%) |  |  |  |  |  |  |
|  |  |  |  |  |  |  |  |  |
| **Age (m)** | 41.7 ± 47.3 | 81.5±77.9 | **p=0.02** |  | 0.993 | 0.006 | 0.982 - 1.004 | 0.234 |

*Significant difference to the rest of the parameters within the group, SE = standard error

**Supplementary Table 3:** CCOS by primary treatment modality

|  | **Discharge** | | |  | **Follow-Up** | | |
| --- | --- | --- | --- | --- | --- | --- | --- |
| CCOS | **improved** | **unchanged** | **worse** |  | **improved** | **unchanged** | **worse** |
| **VP-Shunt** | 85% | 15% | 0% | VP-Shunt | 80% | 10% | 5% |
| **ETV** | 100% | 0% | 0% | ETV | 67% | 22% | 11% |
| **FMD extradural** | 100% | 0% | 0% | FMD extradural | 75% | 25% | 0% |
| **FMD intradural** | 50% | 50% | 0% | FMD intradural | 78% | 11% | 11% |
| **Filum untethering** | 100% | 0% |  | Filum untethering | 100% | 0% | 0% |
| **Cranial vault expansion** | 0% | 100% |  | Cranial vault expansion | 100% | 0% | 0% |
| **Total** | 76% | 18% | 0% | Total | 69% | 14% | 6% |

**Supplementary Table 4:** Baseline parameters by study center

|  | | Center | | | | | | | | | | | | | | | | | | | | | | | | | | | |
| --- | --- | --- | --- | --- | --- | --- | --- | --- | --- | --- | --- | --- | --- | --- | --- | --- | --- | --- | --- | --- | --- | --- | --- | --- | --- | --- | --- | --- | --- |
|  |  | 1 | | 2 | | 3 | | | | 4 | | 5 | | | 6 | | | 7 | | | 8 | | | | 9 | | 10 | | |
|  |  | n | (%) | n | (%) | | n | (%) | n | | (%) | | n | (%) | | n | (%) | | n | (%) | | n | (%) | n | | (%) | | n | (%) |
| Gender | male | 3 | 9.4% | 4 | 12.5% | | 1 | 3.1% | 1 | | 3.1% | | 3 | 9.4% | | 4 | 12.5% | | 3 | 9.4% | | 1 | 3.1% | 9 | | 28.1% | | 3 | 9.4% |
|  | female | 2 | 11.8% | 1 | 5.9% | | 0 | 0.0% | 1 | | 5.9% | | 2 | 11.8% | | 3 | 17.6% | | 0 | 0.0% | | 2 | 11.8% | 3 | | 17.6% | | 3 | 17.6% |
| no preoperative symptoms | no | 4 | 9.1% | 5 | 11.4% | | 1 | 2.3% | 2 | | 4.5% | | 3 | 6.8% | | 7 | 15.9% | | 3 | 6.8% | | 3 | 6.8% | 12 | | 27.3% | | 4 | 9.1% |
|  | yes | 1 | 20.0% | 0 | 0.0% | | 0 | 0.0% | 0 | | 0.0% | | 2 | 40.0% | | 0 | 0.0% | | 0 | 0.0% | | 0 | 0.0% | 0 | | 0.0% | | 2 | 40.0% |
| Chiari typical headaches | no | 5 | 11.6% | 5 | 11.6% | | 1 | 2.3% | 1 | | 2.3% | | 2 | 4.7% | | 6 | 14.0% | | 3 | 7.0% | | 3 | 7.0% | 11 | | 25.6% | | 6 | 14.0% |
|  | yes | 0 | 0.0% | 0 | 0.0% | | 0 | 0.0% | 1 | | 16.7% | | 3 | 50.0% | | 1 | 16.7% | | 0 | 0.0% | | 0 | 0.0% | 1 | | 16.7% | | 0 | 0.0% |
| non-Chiari typical headaches | no | 5 | 11.6% | 5 | 11.6% | | 1 | 2.3% | 2 | | 4.7% | | 5 | 11.6% | | 4 | 9.3% | | 3 | 7.0% | | 1 | 2.3% | 12 | | 27.9% | | 5 | 11.6% |
|  | yes | 0 | 0.0% | 0 | 0.0% | | 0 | 0.0% | 0 | | 0.0% | | 0 | 0.0% | | 3 | 50.0% | | 0 | 0.0% | | 2 | 33.3% | 0 | | 0.0% | | 1 | 16.7% |
| CN deficits | no | 5 | 11.6% | 4 | 9.3% | | 1 | 2.3% | 0 | | 0.0% | | 5 | 11.6% | | 6 | 14.0% | | 3 | 7.0% | | 3 | 7.0% | 10 | | 23.3% | | 6 | 14.0% |
|  | yes | 0 | 0.0% | 1 | 16.7% | | 0 | 0.0% | 2 | | 33.3% | | 0 | 0.0% | | 1 | 16.7% | | 0 | 0.0% | | 0 | 0.0% | 2 | | 33.3% | | 0 | 0.0% |
| Vomiting | no | 5 | 11.4% | 5 | 11.4% | | 1 | 2.3% | 1 | | 2.3% | | 3 | 6.8% | | 7 | 15.9% | | 2 | 4.5% | | 2 | 4.5% | 12 | | 27.3% | | 6 | 13.6% |
|  | yes | 0 | 0.0% | 0 | 0.0% | | 0 | 0.0% | 1 | | 20.0% | | 2 | 40.0% | | 0 | 0.0% | | 1 | 20.0% | | 1 | 20.0% | 0 | | 0.0% | | 0 | 0.0% |
| failure to thrive | no | 5 | 10.4% | 5 | 10.4% | | 1 | 2.1% | 2 | | 4.2% | | 5 | 10.4% | | 6 | 12.5% | | 3 | 6.3% | | 3 | 6.3% | 12 | | 25.0% | | 6 | 12.5% |
|  | yes | 0 | 0.0% | 0 | 0.0% | | 0 | 0.0% | 0 | | 0.0% | | 0 | 0.0% | | 1 | 100.0% | | 0 | 0.0% | | 0 | 0.0% | 0 | | 0.0% | | 0 | 0.0% |
| sleep apnea | no | 5 | 10.6% | 5 | 10.6% | | 1 | 2.1% | 2 | | 4.3% | | 5 | 10.6% | | 7 | 14.9% | | 3 | 6.4% | | 3 | 6.4% | 10 | | 21.3% | | 6 | 12.8% |
|  | yes | 0 | 0.0% | 0 | 0.0% | | 0 | 0.0% | 0 | | 0.0% | | 0 | 0.0% | | 0 | 0.0% | | 0 | 0.0% | | 0 | 0.0% | 2 | | 100.0% | | 0 | 0.0% |
| sensory deficits | no | 5 | 11.4% | 5 | 11.4% | | 0 | 0.0% | 2 | | 4.5% | | 5 | 11.4% | | 6 | 13.6% | | 1 | 2.3% | | 3 | 6.8% | 12 | | 27.3% | | 5 | 11.4% |
|  | yes | 0 | 0.0% | 0 | 0.0% | | 1 | 20.0% | 0 | | 0.0% | | 0 | 0.0% | | 1 | 20.0% | | 2 | 40.0% | | 0 | 0.0% | 0 | | 0.0% | | 1 | 20.0% |
| motor deficits | no | 5 | 11.4% | 4 | 9.1% | | 1 | 2.3% | 2 | | 4.5% | | 5 | 11.4% | | 7 | 15.9% | | 2 | 4.5% | | 3 | 6.8% | 10 | | 22.7% | | 5 | 11.4% |
|  | yes | 0 | 0.0% | 1 | 20.0% | | 0 | 0.0% | 0 | | 0.0% | | 0 | 0.0% | | 0 | 0.0% | | 1 | 20.0% | | 0 | 0.0% | 2 | | 40.0% | | 1 | 20.0% |
| gait disturbance | no | 5 | 11.1% | 5 | 11.1% | | 1 | 2.2% | 1 | | 2.2% | | 5 | 11.1% | | 6 | 13.3% | | 2 | 4.4% | | 2 | 4.4% | 12 | | 26.7% | | 6 | 13.3% |
|  | yes | 0 | 0.0% | 0 | 0.0% | | 0 | 0.0% | 1 | | 25.0% | | 0 | 0.0% | | 1 | 25.0% | | 1 | 25.0% | | 1 | 25.0% | 0 | | 0.0% | | 0 | 0.0% |
| increased head circumference | no | 3 | 10.3% | 3 | 10.3% | | 1 | 3.4% | 2 | | 6.9% | | 5 | 17.2% | | 5 | 17.2% | | 2 | 6.9% | | 2 | 6.9% | 2 | | 6.9% | | 4 | 13.8% |
|  | yes | 2 | 10.0% | 2 | 10.0% | | 0 | 0.0% | 0 | | 0.0% | | 0 | 0.0% | | 2 | 10.0% | | 1 | 5.0% | | 1 | 5.0% | 10 | | 50.0% | | 2 | 10.0% |
| full fontanelle | no | 5 | 11.4% | 4 | 9.1% | | 1 | 2.3% | 2 | | 4.5% | | 5 | 11.4% | | 6 | 13.6% | | 3 | 6.8% | | 2 | 4.5% | 10 | | 22.7% | | 6 | 13.6% |
|  | yes | 0 | 0.0% | 1 | 20.0% | | 0 | 0.0% | 0 | | 0.0% | | 0 | 0.0% | | 1 | 20.0% | | 0 | 0.0% | | 1 | 20.0% | 2 | | 40.0% | | 0 | 0.0% |
| sunset phenomenon | no | 5 | 10.4% | 5 | 10.4% | | 1 | 2.1% | 2 | | 4.2% | | 5 | 10.4% | | 7 | 14.6% | | 3 | 6.3% | | 3 | 6.3% | 11 | | 22.9% | | 6 | 12.5% |
|  | yes | 0 | 0.0% | 0 | 0.0% | | 0 | 0.0% | 0 | | 0.0% | | 0 | 0.0% | | 0 | 0.0% | | 0 | 0.0% | | 0 | 0.0% | 1 | | 100.0% | | 0 | 0.0% |
| vertigo | no | 5 | 10.6% | 5 | 10.6% | | 1 | 2.1% | 2 | | 4.3% | | 5 | 10.6% | | 7 | 14.9% | | 3 | 6.4% | | 2 | 4.3% | 12 | | 25.5% | | 5 | 10.6% |
|  | yes | 0 | 0.0% | 0 | 0.0% | | 0 | 0.0% | 0 | | 0.0% | | 0 | 0.0% | | 0 | 0.0% | | 0 | 0.0% | | 1 | 50.0% | 0 | | 0.0% | | 1 | 50.0% |
| other preop symptoms | no | 2 | 6.9% | 2 | 6.9% | | 1 | 3.4% | 2 | | 6.9% | | 2 | 6.9% | | 6 | 20.7% | | 1 | 3.4% | | 0 | 0.0% | 9 | | 31.0% | | 4 | 13.8% |
|  | yes | 3 | 15.0% | 3 | 15.0% | | 0 | 0.0% | 0 | | 0.0% | | 3 | 15.0% | | 1 | 5.0% | | 2 | 10.0% | | 3 | 15.0% | 3 | | 15.0% | | 2 | 10.0% |
| Main symptoms caused by Chiari | no | 5 | 11.6% | 5 | 11.6% | | 1 | 2.3% | 2 | | 4.7% | | 5 | 11.6% | | 5 | 11.6% | | 0 | 0.0% | | 3 | 7.0% | 12 | | 27.9% | | 5 | 11.6% |
|  | yes | 0 | 0.0% | 0 | 0.0% | | 0 | 0.0% | 0 | | 0.0% | | 0 | 0.0% | | 2 | 33.3% | | 3 | 50.0% | | 0 | 0.0% | 0 | | 0.0% | | 1 | 16.7% |
| Main symptoms caused by hydrocephalus | no | 2 | 18.2% | 0 | 0.0% | | 1 | 9.1% | 1 | | 9.1% | | 2 | 18.2% | | 1 | 9.1% | | 1 | 9.1% | | 0 | 0.0% | 1 | | 9.1% | | 2 | 18.2% |
|  | yes | 3 | 7.9% | 5 | 13.2% | | 0 | 0.0% | 1 | | 2.6% | | 3 | 7.9% | | 6 | 15.8% | | 2 | 5.3% | | 3 | 7.9% | 11 | | 28.9% | | 4 | 10.5% |
| Main symptoms caused by syrinx | no | 5 | 11.9% | 5 | 11.9% | | 0 | 0.0% | 1 | | 2.4% | | 5 | 11.9% | | 6 | 14.3% | | 1 | 2.4% | | 2 | 4.8% | 12 | | 28.6% | | 5 | 11.9% |
|  | yes | 0 | 0.0% | 0 | 0.0% | | 1 | 14.3% | 1 | | 14.3% | | 0 | 0.0% | | 1 | 14.3% | | 2 | 28.6% | | 1 | 14.3% | 0 | | 0.0% | | 1 | 14.3% |
| Main symptoms caused by other | no | 3 | 6.8% | 5 | 11.4% | | 1 | 2.3% | 2 | | 4.5% | | 4 | 9.1% | | 7 | 15.9% | | 2 | 4.5% | | 3 | 6.8% | 11 | | 25.0% | | 6 | 13.6% |
|  | yes | 2 | 40.0% | 0 | 0.0% | | 0 | 0.0% | 0 | | 0.0% | | 1 | 20.0% | | 0 | 0.0% | | 1 | 20.0% | | 0 | 0.0% | 1 | | 20.0% | | 0 | 0.0% |
| no comorbidities | no | 4 | 10.5% | 3 | 7.9% | | 1 | 2.6% | 2 | | 5.3% | | 4 | 10.5% | | 6 | 15.8% | | 3 | 7.9% | | 1 | 2.6% | 10 | | 26.3% | | 4 | 10.5% |
|  | yes | 1 | 9.1% | 2 | 18.2% | | 0 | 0.0% | 0 | | 0.0% | | 1 | 9.1% | | 1 | 9.1% | | 0 | 0.0% | | 2 | 18.2% | 2 | | 18.2% | | 2 | 18.2% |
| Ehlers Danlos | no | 5 | 10.2% | 5 | 10.2% | | 1 | 2.0% | 2 | | 4.1% | | 5 | 10.2% | | 7 | 14.3% | | 3 | 6.1% | | 3 | 6.1% | 12 | | 24.5% | | 6 | 12.2% |
|  | yes | 0 | 0.0% | 0 | 0.0% | | 0 | 0.0% | 0 | | 0.0% | | 0 | 0.0% | | 0 | 0.0% | | 0 | 0.0% | | 0 | 0.0% | 0 | | 0.0% | | 0 | 0.0% |
| Crouzon | no | 5 | 10.9% | 5 | 10.9% | | 1 | 2.2% | 2 | | 4.3% | | 4 | 8.7% | | 7 | 15.2% | | 3 | 6.5% | | 3 | 6.5% | 12 | | 26.1% | | 4 | 8.7% |
|  | yes | 0 | 0.0% | 0 | 0.0% | | 0 | 0.0% | 0 | | 0.0% | | 1 | 33.3% | | 0 | 0.0% | | 0 | 0.0% | | 0 | 0.0% | 0 | | 0.0% | | 2 | 66.7% |
| Pfeiffer | no | 5 | 10.4% | 5 | 10.4% | | 1 | 2.1% | 2 | | 4.2% | | 4 | 8.3% | | 7 | 14.6% | | 3 | 6.3% | | 3 | 6.3% | 12 | | 25.0% | | 6 | 12.5% |
|  | yes | 0 | 0.0% | 0 | 0.0% | | 0 | 0.0% | 0 | | 0.0% | | 1 | 100.0% | | 0 | 0.0% | | 0 | 0.0% | | 0 | 0.0% | 0 | | 0.0% | | 0 | 0.0% |
| Achondroplasia | no | 5 | 10.4% | 5 | 10.4% | | 1 | 2.1% | 2 | | 4.2% | | 5 | 10.4% | | 7 | 14.6% | | 3 | 6.3% | | 3 | 6.3% | 11 | | 22.9% | | 6 | 12.5% |
|  | yes | 0 | 0.0% | 0 | 0.0% | | 0 | 0.0% | 0 | | 0.0% | | 0 | 0.0% | | 0 | 0.0% | | 0 | 0.0% | | 0 | 0.0% | 1 | | 100.0% | | 0 | 0.0% |
| Obesity | no | 5 | 10.4% | 5 | 10.4% | | 1 | 2.1% | 2 | | 4.2% | | 5 | 10.4% | | 7 | 14.6% | | 2 | 4.2% | | 3 | 6.3% | 12 | | 25.0% | | 6 | 12.5% |
|  | yes | 0 | 0.0% | 0 | 0.0% | | 0 | 0.0% | 0 | | 0.0% | | 0 | 0.0% | | 0 | 0.0% | | 1 | 100.0% | | 0 | 0.0% | 0 | | 0.0% | | 0 | 0.0% |
| heart disease | no | 5 | 11.1% | 5 | 11.1% | | 1 | 2.2% | 2 | | 4.4% | | 3 | 6.7% | | 7 | 15.6% | | 3 | 6.7% | | 3 | 6.7% | 10 | | 22.2% | | 6 | 13.3% |
|  | yes | 0 | 0.0% | 0 | 0.0% | | 0 | 0.0% | 0 | | 0.0% | | 2 | 50.0% | | 0 | 0.0% | | 0 | 0.0% | | 0 | 0.0% | 2 | | 50.0% | | 0 | 0.0% |
| Tethered cord | no | 4 | 8.3% | 5 | 10.4% | | 1 | 2.1% | 2 | | 4.2% | | 5 | 10.4% | | 7 | 14.6% | | 3 | 6.3% | | 3 | 6.3% | 12 | | 25.0% | | 6 | 12.5% |
|  | yes | 1 | 100.0% | 0 | 0.0% | | 0 | 0.0% | 0 | | 0.0% | | 0 | 0.0% | | 0 | 0.0% | | 0 | 0.0% | | 0 | 0.0% | 0 | | 0.0% | | 0 | 0.0% |
| Down's Syndrome | no | 5 | 10.2% | 5 | 10.2% | | 1 | 2.0% | 2 | | 4.1% | | 5 | 10.2% | | 7 | 14.3% | | 3 | 6.1% | | 3 | 6.1% | 12 | | 24.5% | | 6 | 12.2% |
|  | yes | 0 | 0.0% | 0 | 0.0% | | 0 | 0.0% | 0 | | 0.0% | | 0 | 0.0% | | 0 | 0.0% | | 0 | 0.0% | | 0 | 0.0% | 0 | | 0.0% | | 0 | 0.0% |
| VACTERL | no | 5 | 10.2% | 5 | 10.2% | | 1 | 2.0% | 2 | | 4.1% | | 5 | 10.2% | | 7 | 14.3% | | 3 | 6.1% | | 3 | 6.1% | 12 | | 24.5% | | 6 | 12.2% |
|  | yes | 0 | 0.0% | 0 | 0.0% | | 0 | 0.0% | 0 | | 0.0% | | 0 | 0.0% | | 0 | 0.0% | | 0 | 0.0% | | 0 | 0.0% | 0 | | 0.0% | | 0 | 0.0% |
| other comorbidity | no | 1 | 4.8% | 3 | 14.3% | | 0 | 0.0% | 1 | | 4.8% | | 3 | 14.3% | | 3 | 14.3% | | 1 | 4.8% | | 2 | 9.5% | 3 | | 14.3% | | 4 | 19.0% |
|  | yes | 4 | 14.3% | 2 | 7.1% | | 1 | 3.6% | 1 | | 3.6% | | 2 | 7.1% | | 4 | 14.3% | | 2 | 7.1% | | 1 | 3.6% | 9 | | 32.1% | | 2 | 7.1% |
| Papillar edema | no | 2 | 6.9% | 5 | 17.2% | | 1 | 3.4% | 1 | | 3.4% | | 5 | 17.2% | | 0 | 0.0% | | 3 | 10.3% | | 2 | 6.9% | 5 | | 17.2% | | 5 | 17.2% |
|  | yes | 1 | 25.0% | 0 | 0.0% | | 0 | 0.0% | 0 | | 0.0% | | 0 | 0.0% | | 0 | 0.0% | | 0 | 0.0% | | 1 | 25.0% | 2 | | 50.0% | | 0 | 0.0% |
|  | unknown | 2 | 13.3% | 0 | 0.0% | | 0 | 0.0% | 1 | | 6.7% | | 0 | 0.0% | | 7 | 46.7% | | 0 | 0.0% | | 0 | 0.0% | 5 | | 33.3% | | 0 | 0.0% |
